# 4-(Nitrophenylsulfonyl)piperazines mitigate radiation damage to multiple tissues

**DOI:** 10.1371/journal.pone.0181577

**Published:** 2017-07-21

**Authors:** Ewa D. Micewicz, Kwanghee Kim, Keisuke S. Iwamoto, Josephine A. Ratikan, Genhong Cheng, Gayle M. Boxx, Robert D. Damoiseaux, Julian P. Whitelegge, Piotr Ruchala, Christine Nguyen, Prabhat Purbey, Joseph Loo, Gang Deng, Michael E. Jung, James W. Sayre, Andrew J. Norris, Dörthe Schaue, William H. McBride

**Affiliations:** 1 Department of Radiation Oncology, University of California at Los Angeles, Los Angeles, California, United States of America; 2 Department of Microbiology, Immunology, and Molecular Genetics, University of California at Los Angeles, Los Angeles, California, United States of America; 3 Molecular Screening Shared Resource, University of California at Los Angeles, Los Angeles, California, United States of America; 4 Pasarow Mass Spectrometry Laboratory, University of California at Los Angeles, Los Angeles, California, United States of America; 5 Department of Chemistry and Biochemistry, University of California at Los Angeles, Los Angeles, California, United States of America; 6 School of Public Health, Biostatistics and Radiology, University of California at Los Angeles, Los Angeles, California, United States of America; 7 BCN Biosciences, LLC, Pasadena, California, United States of America; ENEA Centro Ricerche Casaccia, ITALY

## Abstract

Our ability to use ionizing radiation as an energy source, as a therapeutic agent, and, unfortunately, as a weapon, has evolved tremendously over the past 120 years, yet our tool box to handle the consequences of accidental and unwanted radiation exposure remains very limited. We have identified a novel group of small molecule compounds with a 4-nitrophenylsulfonamide (NPS) backbone in common that dramatically decrease mortality from the hematopoietic acute radiation syndrome (hARS). The group emerged from an in vitro high throughput screen (HTS) for inhibitors of radiation-induced apoptosis. The lead compound also mitigates against death after local abdominal irradiation and after local thoracic irradiation (LTI) in models of subacute radiation pneumonitis and late radiation fibrosis. Mitigation of hARS is through activation of radiation-induced CD11b^+^Ly6G^+^Ly6C^+^ immature myeloid cells. This is consistent with the notion that myeloerythroid-restricted progenitors protect against WBI-induced lethality and extends the possible involvement of the myeloid lineage in radiation effects. The lead compound was active if given to mice before or after WBI and had some anti-tumor action, suggesting that these compounds may find broader applications to cancer radiation therapy.

## Introduction

The threat level for exposure of large numbers of people to ionizing radiation has been significantly elevated following the worldwide rise in terrorism. Potentially devastating scenarios include addition of radioactive materials to food or drink, explosive devices containing radioactive sources, or more sophisticated nuclear explosives. Nuclear disasters such as at Fukushima, Chernobyl, and Goiania further fuel public concern. Several governmental agencies have acknowledged the paucity of countermeasures for radiation damage, prompting efforts to develop treatments that are effective when started at least one day after exposure. Since radiation-induced cell death and tissue damage are classically thought of as consequences of free radical generation, DNA damage repair, and rapid apoptosis; events that are largely over within hours of exposure, delayed treatment shifts the spotlight to downstream processes that interpret and amplify initial radiation-induced DNA damage responses. Notwithstanding this stringent requirement, a number of compounds have been identified that mitigate lethality from acute radiation syndromes (ARS) in preclinical models [1–10]; although structure-activity relationships and pathways to mitigation are generally obscure and agents active against the broad spectrum of possible radiation syndromes are lacking.

A unique group of chemically similar, broadly acting radiation mitigators emerged from our screens of small molecule chemical libraries for agents that blocked rapid apoptotic death of irradiated lymphocytes when added 1 hr after irradiation of cells in vitro. Remarkably, these compounds mitigated lethal hARS when given to mice 24hrs after whole body irradiation (WBI). The lead compound was additionally effective as a mitigator of lethal intestinal ARS, subacute radiation pneumonitis and late pulmonary fibrosis, and radioprotected mice when given before WBI. At least for hARS, there is an absolute requirement for CD11b^+^Ly6G^+^Ly6C^+^ myeloid cells. The survival advantage conferred by acute mitigation of radiation damage is long lasting, and there was no increase in radiation-induced cancers (Schaue, in preparation). These compounds have low toxicity, and some anti-tumor action, suggesting that they may also be of use in the broader context of radiotherapy for cancer.

## Materials and methods

UCLA's IACUC-approved protocols and NIH guidelines and defined criteria for premature euthanasia were adhered to. Animal health was monitored at least daily and irradiated mice were followed more closely 2–3 times, as needed. Body weight was assessed twice per week. Euthanasia was by exposure to carbon dioxide confirmed by cervical dislocation. Animals were euthanized when tumors reached 1.3 cm in any diameter. No animals showed any signs of illness following tumor formation as the experiments were terminated prior to pain and suffering. Other criteria for premature euthanasia in the context of radiation included weight loss (up to 20%), labored breathing, decreased mobility, difficulties reaching food or water, hunching, prolonged lethargy, bloody or excessive diarrhea lasting 2 days, inability to remain upright, loss of body condition (BCS from 3 to 2). There were no unexpected deaths due to experimental procedures or other causes and without euthanasia. The experiments were approved under ARC number #1999–173.

### High throughput screening and drugs

The HTS assay has been described previously [10]. Cells from the CD4+CD8+ murine TIL1 lymphocytic line [11] were irradiated in vitro with 2Gy in MEM medium with 10% fetal calf serum and 1hr later, 85,000 chemically diversified compounds from the ChemBridge DIVERSet (San Diego, CA) or the Asinex or Asinex Targeted (Moscow, Russia) libraries were individually added at 10 uM final concentration in 1% DMSO using an automated Biomek FX Workstation (Beckman Coulter, Fullerton, CA). Viability was assessed at 24hrs by ATP production (ATPlite, Perkin-Elmer, MA). Compounds that increased viability to >130% of irradiated (diluent) controls (100%) were verified by retesting at varying concentrations in ATP-Lite and Annexin/P.I. assays.

For in vivo assays, NPS or NPSP compounds were obtained from ChemBridge (San Diego, CA) or synthesized in house. Purity and stability were assessed by NMR.

### Similarity and substructure analyses

Data were mined on a Collaborative Drug Discovery vault platform (CDD^™^, Burlingame, CA) and maximal common substructuring (Chemaxon, Boston, MA) was performed for the NPS and NPSP compounds. The entire library was ranked according to its structural similarity to a referenced hit based upon the Tanimoto coefficient, excluding coefficients < 0.7. Hits and non-hits within the library with similar structure were identified and a substructure analysis performed to determine minimal core moieties.

### Mouse irradiation

C3Hf/Sed//Kam or C57Bl/6 gnotobiotic male or female mice were bred in our Radiation Oncology AAALAC-accredited facility and utilized at a body weight of 28gms (with 1S.D.<1gm; 9-12wks of age). IACUC-approved protocols and NIH guidelines and defined criteria for premature euthanasia were followed.

WBI was performed using an AEC Gamma Cell 40 cesium irradiator (Cs-137) at a dose rate of around 60 cGy/min with unanesthetized mice in a well-ventilated Lucite box able to move around during irradiation. The LD70/30 estimates derived from probit analyses were for our C3H mice 7.725Gy, for our C57Bl/6 mice 8.509Gy. In order to determine the dose modification factor following mitigation male C3H mice were whole-body irradiated with a range of 5 different doses (n = 8 per group) and 24h later treated s.c. with 5mg/kg compound #5 or 75mg/kg compound #10, repeated daily for 5dys. The shift in radiation mortality was determined according to Probit analysis.

Partial body irradiations used 300kV X-rays (Gulmay, Surrey, UK) with anesthesia for better collimation with Cerrobend (1cm) shielding of other body parts. Mice were anaesthetized with an i.p. injection of 80mg/kg Ketamine (Putney, NADA#200–073) and 4mg/kg Xylazine (AnaSed, NADA# 139–236; Lloyd labs #4811), which was sufficient to maintain good anesthesia for 30mins under regular breathing. Anesthetized mice were positioned on a platform with cerrobend jig to shield the rest of the body. Dosimetry used a Capintec ionization chamber calibrated to NIST standards and film (GAFCHROMIC EBT2, International Specialty Products, Wayne, NJ) to check that deviations in the field uniformity is <5%.

Mice were monitored for at least 30 days and defined criteria for humane euthanasia was used as an endpoint. To allow more valid comparisons to be made between compounds, they were dissolved in 15μl DMSO and suspended in 1ml of 1% Cremophor EL in water for administration in 0.2ml volumes. Other vehicle formulations were tested as noted in the text, but this amount of Cremophor did not significantly alter the response to WBI. All mice, including controls, mice received the same diluent as the experimental groups.

### Tumor model

Mice were injected with 5x10^4^ Lewis Lung carcinoma cells i.v. and treated with compound #5 (20mg/kg s.c.) or diluent on days 4–8 and given 4Gy LTI on days 5–7. Lungs were harvested on day 14 and the lung tumor nodules counted after staining in Bouin’s solution.

### Immune correlates

Bone marrow-derived macrophages (BMDMs) were derived by 7 days culture of marrow cells in medium containing 10% FBS and CSF-1 conditioned medium [12]. The serum concentration was reduced to 2% FBS 16h before stimulation with LPS for 1h, treatment with drug and incubation for another 5h (6h total with LPS). Total cellular RNA was isolated by trizol and cDNA synthesized using iScript from BioRad. Gene expression was measured by qPCR and analyzed using the standard curve method, normalized to L32.

Peritoneal macrophages (PMs) were induced by i.p. injection of 150 mg MIS416 [13] (Innate Immunotherapeutics, Auckland, NZ) and harvested on day 4 by peritoneal wash out with PBS. Treatments were given as stated in the text. Culture supernatants were harvested at 24hrs and tested for cytokines. Cytokine multi-array cytokine assays were from RayBiotech (Norcross, GA) and anti-TNF- α assays from AbCam (Cambridge, MA).

Endogenous CFU-S in spleens from mice were counted 10dys after the stated WBI doses and staining in Bouin’s solution.

Flow cytometry used a BD Fortessa LSR machine with labeled antibody panels from BD, San Diego. Anti-Ly6 depletion antibodies (clone 1A8) were from Bio-XCell (West Lebanon, NH) [14] and were given to mice i.p. in 300μg doses every 2 days for 10 days starting 1 hr before WBI (days 0, 2, 4, 6).

Intracellular nitric oxide and ROS production was assessed using commercially available OxiSelect fluorescent assays (Cell Biolabs, San Diego). These were performed using DC2.4 dendritic cells that resist radiation-induced apoptosis [15].

### Pharmacokinetic analyses

Pharmacokinetic data were obtained from plasma samples at various times after a single s.c. injection using ultra-performance liquid chromatography (UPLC) coupled with selected reaction monitoring mass spectrometry (SRM) on triple-quadrupole instruments. Estimates of concentration were obtained using spiked samples of known concentration. PK values were obtained using SummitPk software to calculate C_max_ and T_1/2_.

### Statistics

Kaplan-Meier plot with log rank statistics were used to test for significance in survival differences. Probit analyses were performed using SPSS v20 software and NCSS PASS 13 Power Analysis and Sample Size Software, Kaysville, Utah was used for power analysis. Analysis of variance procedures were performed on all other data with Brown-Forsythe test where homogeneity of variance assumptions were not met. Multiple comparisons procedures using Sidak were also performed. The Kruskal-Wallis non-parametric test was performed with less stringent assumptions for some data distributions. Significance was assessed at the 5% level using SPSS v20 software (IBM SPSS Statistics, Armonk, NY).

## Results

### HTS for mitigators of radiation-induced lymphocyte apoptosis

**Fig 1:**
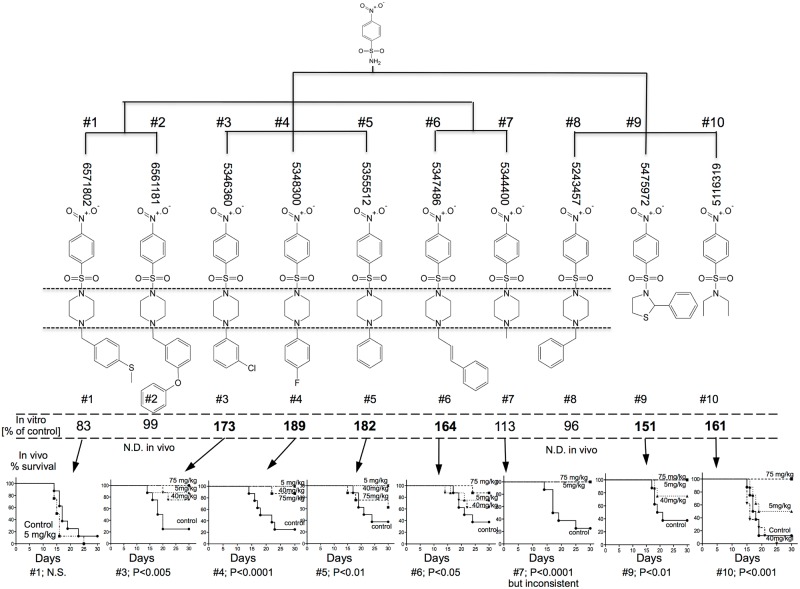
4-Nitrophenylsulfonamides effectively mitigate radiation damage in vitro and in vivo. NPSP (#1–8) and NPS (#9–10) chemical structures with ChemBridge nomenclature arranged by maximal common substructuring. The data underneath each compound refers to % viability of TIL1 lymphocytic cells at 24 hrs, compounds being added at 10μM to TIL1 cells 1 hr after 2Gy irradiation. Viability was assessed by ATPLite production at 24 hrs and is shown relative to 100% of irradiated controls, with >130% (bold) being taken as a significant increase (>3S.D. above control mean). There were no significant toxic or stimulatory effects when added to non-irradiated cells. All except #1 and #8 were tested in vivo (bottom graphs). They were injected in 1% Cremophor s.c. into C3H male mice (8 per group) starting 24 hrs after 7.725Gy WBI (LD70/30 estimate), daily for 5 days. Survival to the day 30 endpoint is expressed using a Kaplan-Meier plot with log rank statistics.

85,000 small molecules from chemical libraries were added in an HTS format to pre-irradiated (2Gy) TIL1 lymphocytic cells that are sensitive to radiation apoptosis. Those compounds that increased viability at 24hrs to >130% of irradiated diluent controls (100%) in an ATP-Lite assay were verified as “hits” if they blocked radiation-induced apoptosis in an annexin V/PI flow cytometry assay (data not shown). Four 4-(nitrophenylsulfonyl)piperazines (NPSP) (Fig 1: #3–6) and two 4-nitrophenylsulfonamides (NPS) (Fig 1; #9, 10) emerged at the top of 23 “hits”. Data mining by maximal common substructuring (Chemaxon, Boston, MA) of the libraries identified 4 additional structurally similar molecules that failed to prevent radiation-induced apoptosis in vitro (Fig 1; #1, 2, 7, 8).

### Effective mitigation of radiation syndromes in mice

**Fig 2:**
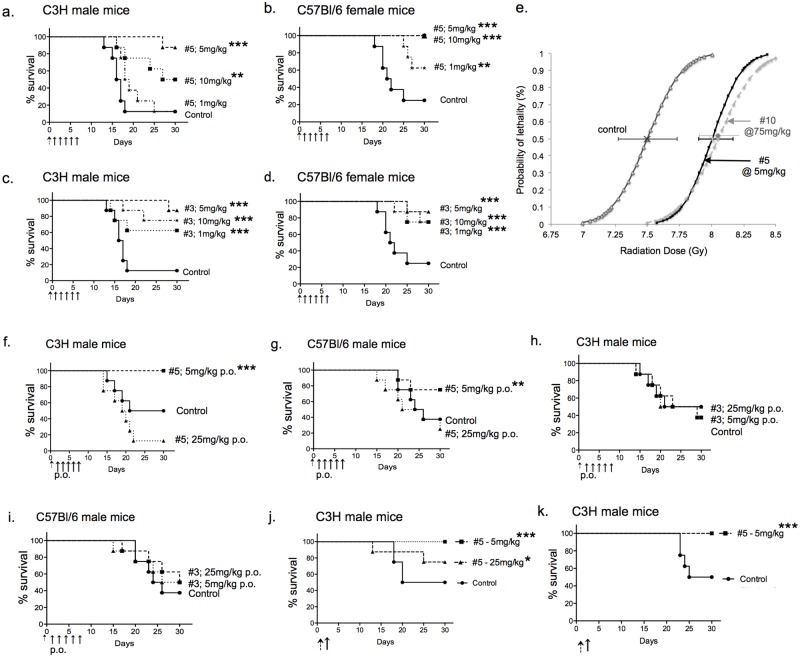
Efficacy of compounds in different conditions. Compounds #5 (a, b) and #3 (c, d) in C3H male (a, c) and C57Bl/6 female (b, d) mice with 5mg/kg given s.c. on day 1 and daily for 5 days. This dose is generally superior to 10 and 1mg/kg (this figure), and 75 and 25mg/kg (Fig 1) for mitigating hARS after WBI (LD70/30 estimated doses used for the different strains). (e) Male C3H mice treated s.c. for 5dys with 5mg/kg compound #5 or 75mg/kg compound #10 have increased resistance to hARS. LD50/30 control = 7.5Gy (95% c.l. = 7.34–7.67); # 5 LD50/30 = 8.0Gy (95% c.l. = 7.9–8.2); #10 LD50/30 = 8.2Gy (95% c.l. = 8.07–8.58). Compound #5 given by gavage at 5mg/kg for 5dys mitigates hARS (LD70/30) in C3H (f) and C57Bl/6 (g) male mice whereas compound #3 does not (h, i). A single s.c. injection of 5mg/kg of compound #5given 24hrs after WBI (LD70/30) mitigates against hARS in C3H mice (j). (k) Compound #5 given 18hrs before WBI (LD70/30 estimated dose of 7.725Gy) as a single 5mg/kg s.c. injection radioprotects C3H male mice from hARS. (*p<0.05, **p<0.01, ***p<0.001).

Mortality within 10-30dys of WBI with doses in the range of 6-10Gy is established to be due to acute hematopoietic syndrome (hARS) [16]. Eight of the compounds in Fig 1 were tested for their ability to mitigate hARS in C3H male mice receiving WBI doses known to cause around 70% mortality (LD70/30). Differences in solubility and pharmacokinetics were minimized a) by suspending compounds in 1% Cremophor and b) by giving daily s.c. injections for 5dys starting at 1dy. Survival was dramatically improved by all 6 of the in vitro active anti-apoptotic compounds (Fig 1). Mortality was not seen until 13dys after WBI, indicating that toxicity, infection, and intestinal damage, which are generally expressed earlier after exposure, were not influences. The cluster containing #3, 4, and 5 were most effective at 5mg/kg, which was generally superior to 75, 40, 10, 2, or 1 mg/kg (Figs 1 and 2a–2d). This dose-responsiveness was not due to toxicity, but rather is an inherent property of these drugs. In contrast, compound #10, which lacks piperazine, was effective in vivo only at 75mg/kg, #1 was inactive in vitro and in vivo, while #7 gave inconsistent data (not shown). The reason for inactivity of some of the related compounds may be inferred indirectly from published X-ray crystal analysis of 4-phenyl-piperazine-1-sulfonamide, which shows 2 molecules vis-á-vis in a highly cohesive antiparallel orientation [17]. While this molecule is not identical to ours, it is sufficiently similar to suggest that variation in biological activities within this group of compounds may be best explained by cohesive stacking. Active mitigators significantly increased the mean survival time (MST) during hARS from 17dys (N = 246 mice) to 18.5dys (N = 401 mice; P<0.02 log rank test). This is consistent with the observation that the MST for any given radiation syndrome is inversely related to dose [16, 18, 19] and that the mitigating action of these agents can in reality be interpreted in terms of radiation dose modification (Fig 2e). Increased survival due to mitigators was lasting with 50% of treated mice alive at 1yr compared to 11% of controls (data not shown).

**Fig 3:**
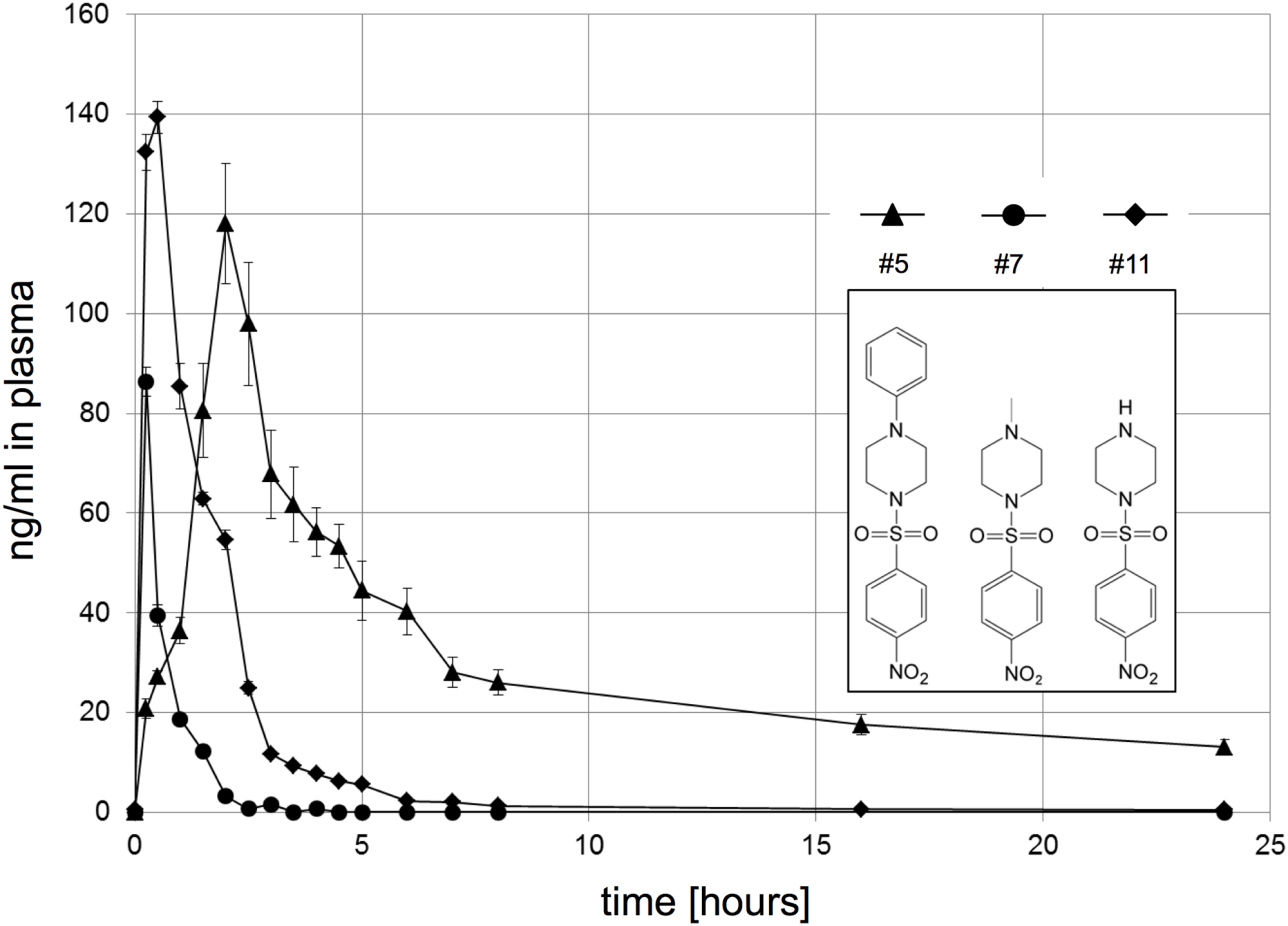
Pharmacokinetics. Pharmacokinetic analysis of compound #5 (triangles) in serum by LC/MS following a single 5 mg/kg s.c. dose showed a drug profile with a C_max_ (obs) of 0.12 μg/mL, a T_max_ of 2h, a half-life of 9h, AUC (area) 0.41 μg-hr/mL and CL (expo) 0.37ml/hr. Persistence was superior to #7 (circles) and #11 (diamonds) (which was synthesized specially, suggesting the phenyl group improves pharmacokinetic availability. Note: This lack of persistence of #7 might explain its variable performance in vivo as a mitigator.

Compounds #3 and 5 were designated as leads based on their potency at low dosage. Both were effective against hARS in female as well as male C3H and C57Bl/6 mice using the same drug and LD70/30 radiation dosing schedules (Fig 2a–2d). Compound #5 but not #3 was effective if given by gavage to either C3H or C57Bl/6 mice (Fig 2f–2i), or as a single s.c. injection of 5mg/kg given at 24hrs (Fig 2j); but less so at 48hrs, and lesser still at 72hrs (Not shown). Compound #5 also protected mice from hARS if given 18hrs before WBI (Fig 2k) and displayed a good pharmacokinetic drug profile (Fig 3) with brain tissue penetration indicated by MALDI-MSI (not shown). There was no evidence of toxicity in any of these experiments, and neither compound #3 nor 5 at a s.c. dose of 200 mg/kg (40 times optimal) caused C3H mice to lose weight, alter differential venous blood cell counts, or gross pathology. The incidence of cancer at 1 year in over 700 mitigated mice was low (<1%) and no higher than in mice surviving LD70/30 doses of WBI without intervention (not shown).

**Fig 4:**
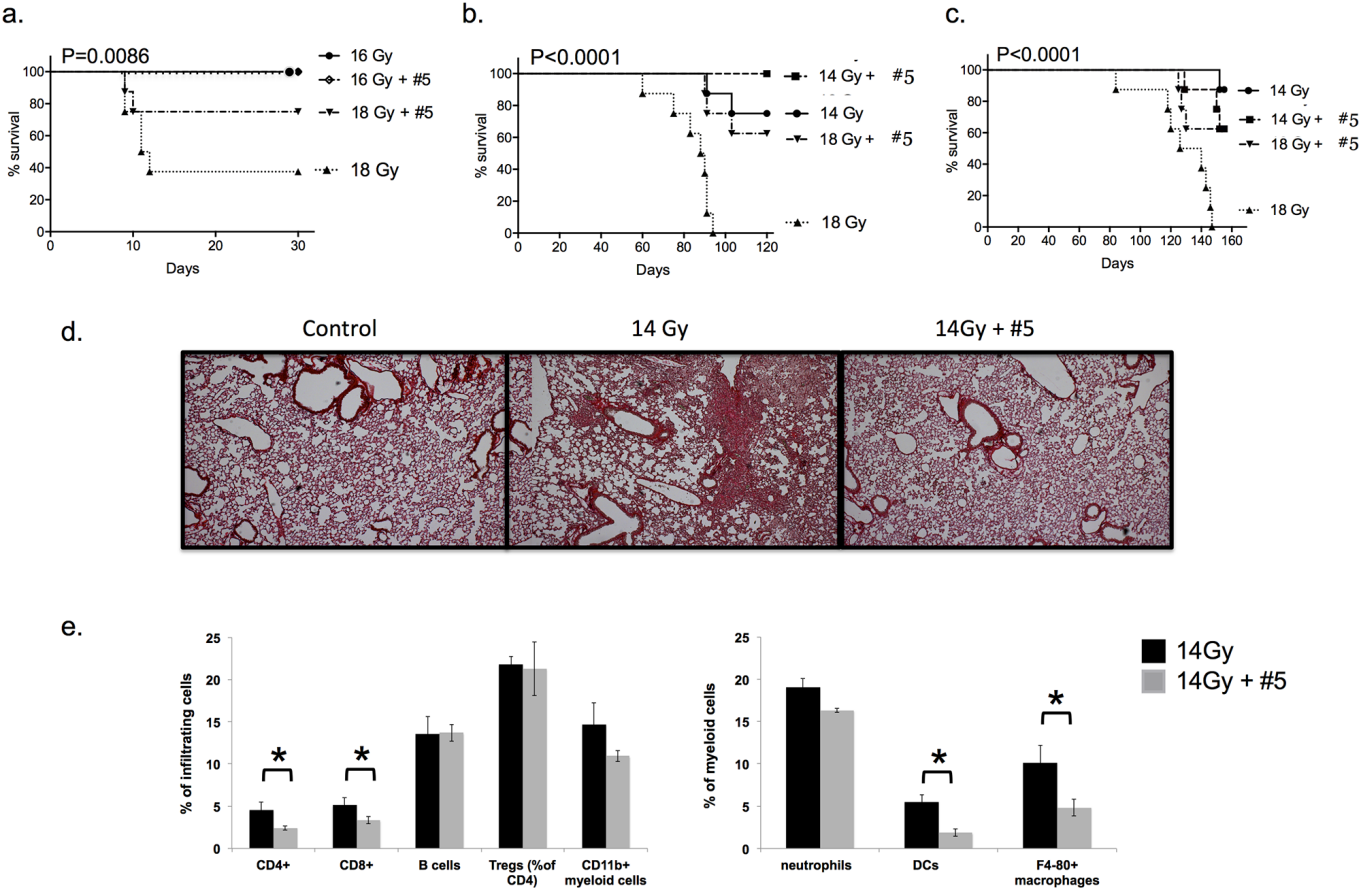
Intestinal and lung damage following radiation exposure can be significantly improved with 4-nitrophenylsulfonamides. (a) Compound #5 (5mg/kg daily x5 s.c.) mitigates lethality from intestinal ARS following 18Gy local abdominal irradiation. (b) Compound #5 (5mg/kg daily x5 s.c.) mitigates lethality from radiation-induced pneumonitis in C3H mice after 18 and 14Gy LTI. (c) Compound #5 (5mg/kg daily x5 s.c.) mitigates lethality from radiation-induced fibrosis in C57Bl/6 mice following 18Gy LTI. Note the differences in time to lethality for these endpoints. Kaplan-Meier with log rank statistics. (d) Picro Sirius Red staining of lungs of C57Bl/6 mice 156 days after LTI with (e) flow cytometric analysis of inflammatory infiltrate. (*p<0.05)

Remarkably, compound #5 given in the standard schedule (5mg/kg s.c. daily for 5 days) mitigated not only hARS but also intestinal ARS after local abdominal irradiation in C57Bl/6 mice, which classically [16] manifests between 7 and 12 days (Fig 4a). Mortality due to subacute pneumonitis in C3H mice and late fibrosis in C57Bl/6 mice, which are classically expressed at 2–3 months and at 4–6 months, respectively, after local thoracic irradiation (LTI) were also mitigated (Fig 4b and 4c). These are standard, strain-specific endpoints for different forms of pulmonary radiation damage [20]. Lungs of C57Bl/6 LTI mice treated with compound #5 showed less fibrosis at 156 days and less myeloid cell infiltrate, particularly macrophages and dendritic cells (Fig 4d and 4e, p<0.05).

### The role of myeloid cells

**Fig 5:**
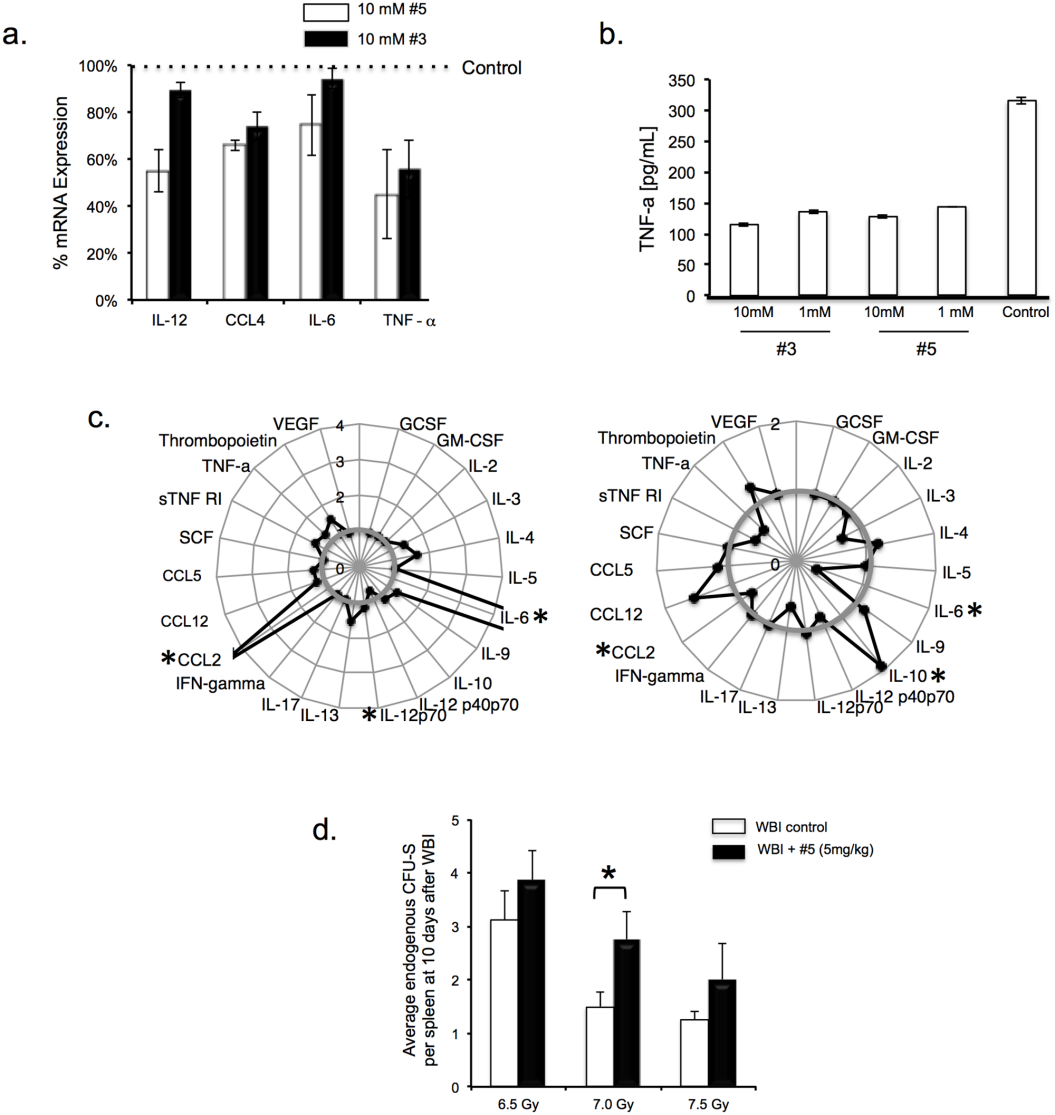
Successfully mitigated mice show favorable immune reconstitution and inflammatory rebalancing. (a) mRNA levels of various cytokines assessed by RT-PCR in bone marrow derived macrophages treated with 10μM #3 or #5 1 hr after 100ng/ml LPS and assayed at 6hrs. All except IL-6 were significantly decreased (P<0.05). (b) TNF-α production at 24hrs by inflammatory peritoneal exudate cells treated in vitro with 100ng/ml LPS followed at 1hr by 1 or 10 μM #3 or #5 or diluent. (c) Bone marrow cells were removed from C3H mice 30hrs after WBI (LD70/30–7.725Gy) or sham irradiated, with compound #5 or diluent given 6 hrs before harvest. After overnight culture, supernatants were tested for cytokines by multi-arrays. The spider plots show changes after WBI alone (left) compared to control (value = 1 in grey) and WBI plus compound #5 (right) compared to WBI alone (value = 1 in grey). (d) Compound #5 (5mg/kg s.c. at 24 hrs after WBI) increases the number of endogenous CFU-S per spleen at day 10 after the WBI. (*p<0.05)

These anti-apoptotic compounds are also anti-inflammatory, which may impact the proliferation and development of hematopoietic and other stem and progenitor cells (HSPC/SPC) [21–24]. Murine bone marrow-derived macrophages or inflammatory peritoneal exudate cells (PEC) from C3H mice, treated for 1hr in vitro with lipopolysaccharide (LPS) prior to addition of compound #3 or #5 had decreased expression of mRNA for TNF-α and other bona-fide pro-inflammatory molecules at 6hrs (Fig 5a), and secreted less TNF-α protein over 24hrs (Fig 5b). Nitric oxide activity was also decreased (not shown). Cytokine production was reprogrammed in a more complex in vivo setting. Bone marrow cells isolated from WBI mice (30hrs after LD70/30) treated with compound #5 (at 24hrs; 5mg/kg s.c.) and cultured overnight in vitro had blunted expression of pro-inflammatory cytokines, such as IL-6, CCL2, and TNF-α and increased anti-inflammatory IL-10 levels (Fig 5c right). Endogenous splenic colonies (CFU-S) were enhanced 10dys post-WBI reaching statistical significance at the 7Gy dose level (Fig 5D).

**Fig 6:**
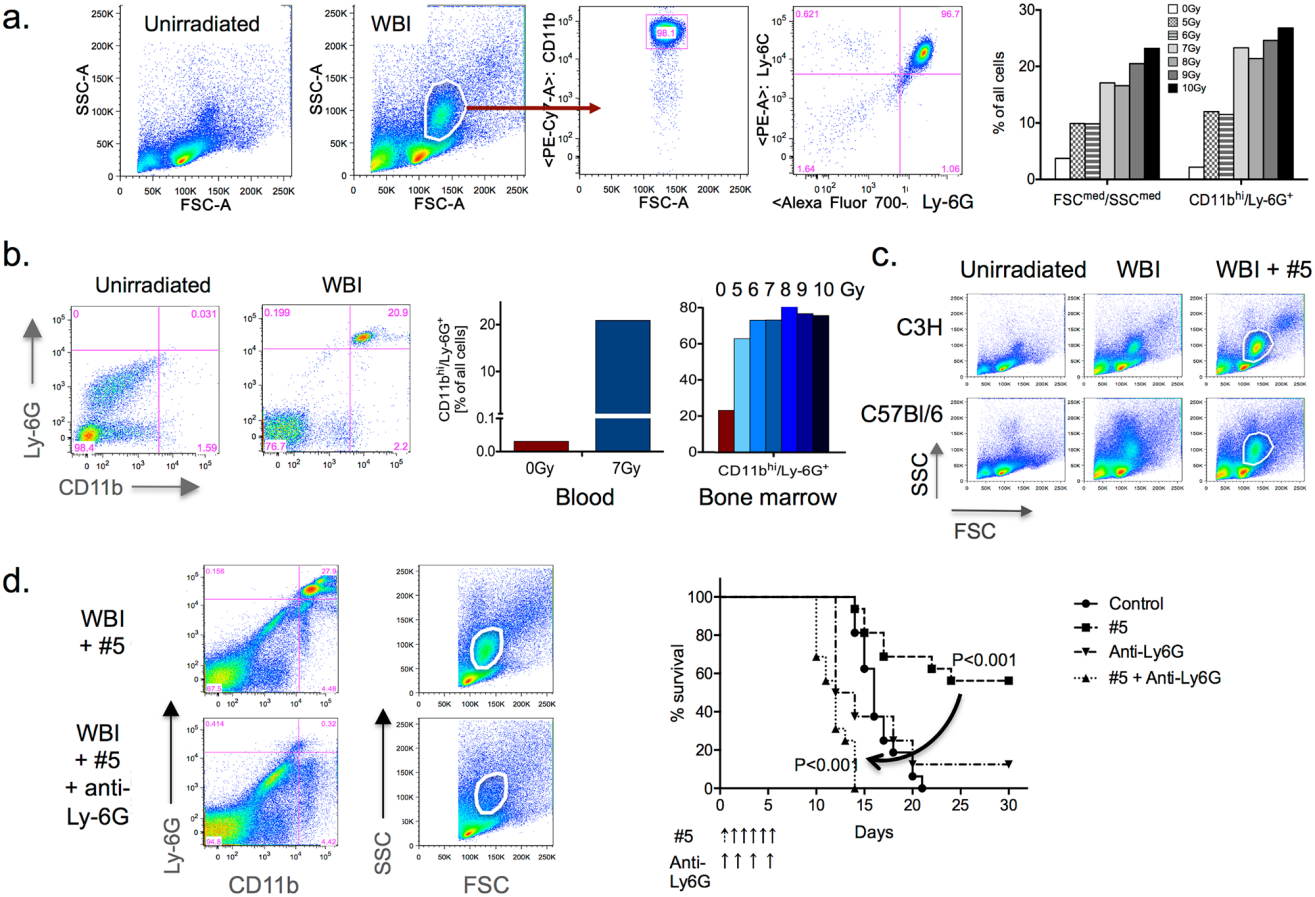
Irradiated mice have a systemic surge in immature myeloid cells that is essential for mitigation. (a) The emergence of a CD11b^+^Ly6G^+^Ly6C^+^ population of immature myeloid cells (middle) in the spleens of mice that are easily distinguishable by forward and side scatter (left) in flow cytometry. Proportional increases as a % of all cells are dose dependent (right), which is in part due to loss of other cells and in part mobilization as few of these cells are present in peripheral organs (see control). (b) The same population appears in the blood (left and middle) and bone marrow (right), where it normally represents 20% of all cells. In blood, where it is normally absent, it reaches levels of 20% of all white cells 30hrs after WBI. (c) Treatment with compound #5 (5mg/kg once) at 24hrs after WBI of C3H or C57Bl/6 mice (LD70/30 estimated dose) increases the CD11b^+^Ly6G^+^Ly6C^+^ representation in the spleen (shown) and other organs (not shown). (d) Treatment of mice with anti-Ly6G removes the CD11b^+^Ly6G^+^Ly6C^+^ population (left) and abolishes activity of mitigator #5 (right).

CFU-S colonies that originate from myeloerythroid-restricted progenitors are known to protect against WBI-induced lethality [25], presumably by allowing time for more primitive stem cells to develop. These are likely related to cells of the promyelocytic and neutrophilic myelocytic lineage that are found in the blood of all species studied within hours of WBI in the lethal range [16]. These are also thought to have a protective role in radiation injury [19], but have not been well characterized. We used flow cytometry to demonstrate that CD11b^+^Ly6G^+^Ly6C^+^ triple-positive immature myeloid cells that pre-exist in bone marrow but are essentially absent from blood, spleen, and other tissues, increase dramatically in the blood and spleen within 6hrs of WBI, peaking by 30hrs when they constituted >25% suof all cells (Fig 6a and 6b). Mitigators such as #5 consistently and reproducibly enhanced the size of the CD11b^+^Ly6G^+^Ly6C^+^ population in the spleen, blood, and bone marrow in both C3H and C57Bl/6 mice after WBI (Fig 6c). Notably, eliminating this population in WBI mice with an anti-Ly6G antibody completely abolished hARS mitigation by these compounds (Fig 6d). The role of these immature myeloid cells in naturally protecting mice against lethality after WBI was evident as anti-Ly6G antibody hastened death in vehicle-treated, WBI mice by about 3 days (Fig 6d).

**Fig 7:**
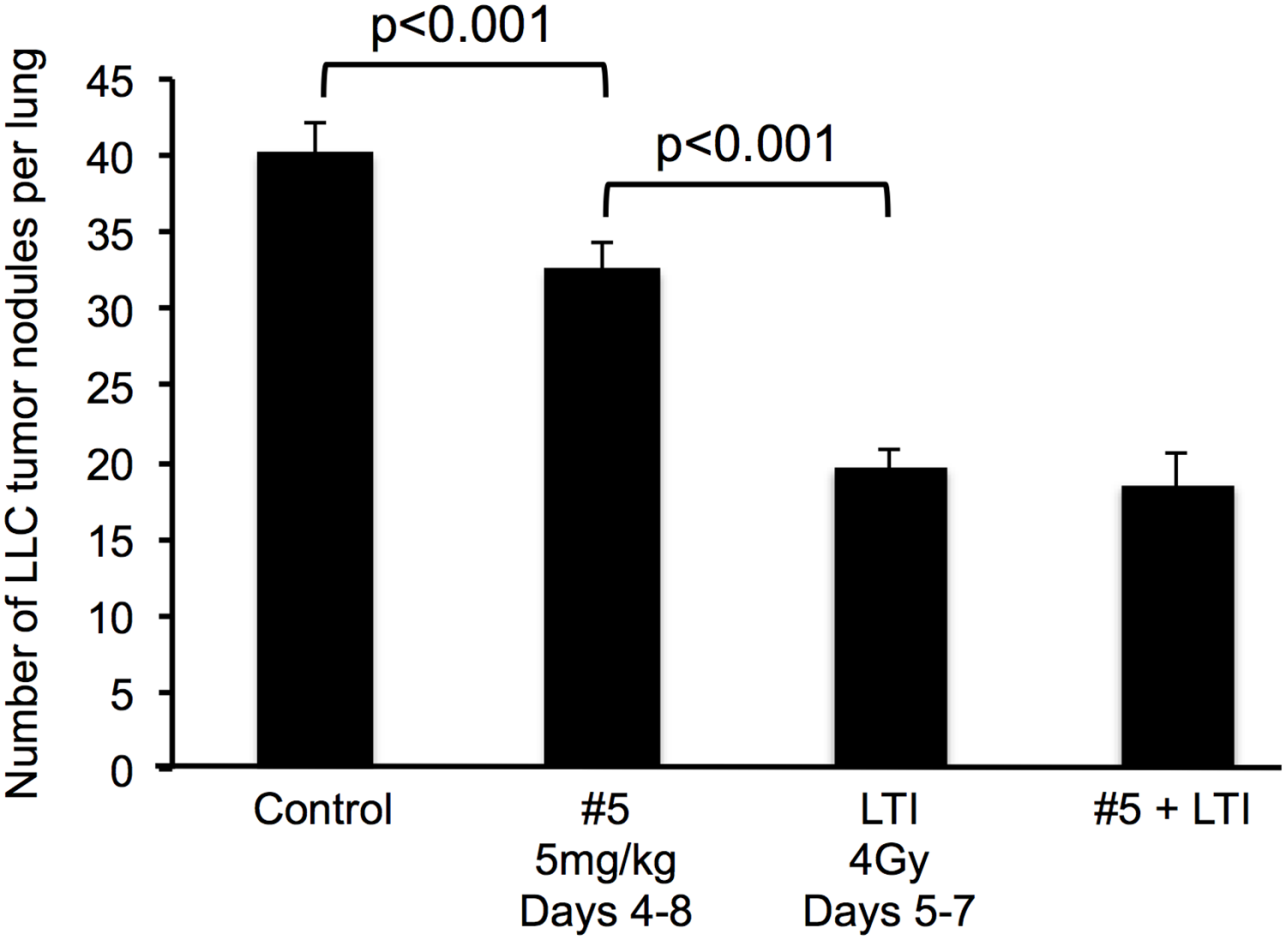
Lung tumors are not protected from radiation damage by the NSPS mitigator #5. Mice were injected with 5x10^4^ Lewis Lung carcinoma (LLC) cells i.v., treated with compound #5 (20mg/kg s.c.) or diluent on days 4–8 and given 0 or 4Gy LTI on days 5–7. Lungs were harvested on day 14 and the lung tumor nodules counted after staining in Bouin’s solution.

Finally, while these mitigators are aimed at use in a radiological disaster, their effectiveness and minimal toxicity beg the question if they will be of broader applicability. Indeed, compound #5 given to mice bearing orthotopic Lewis lung carcinoma (LLC) on days 4–8, with or without LTI (4Gy/day on days 5–7) (Fig 7) did not protect the tumor or stimulate its growth; in fact it had anti-tumor activity. These dual opposing effects on normal and tumor tissues are perhaps not surprising given the role of immature myeloid cells in cancer radiotherapy [26].

## Discussion

While thiol-based radioprotectors, given at the time of irradiation, have classically been used to show the importance of free radicals in radiation damage, no similar group of small molecules have been developed that are active given 24hrs after exposure and in so many radiation syndromes.

In general, sulfonamides and piperazines are very common elements of clinically used drugs. However, the biological literature directly relevant to the drugs described here is remarkably sparse. Pifithrin-μ (2- phenylethynesulfonamide) can protect mice from lethal doses of ionizing radiation [27, 28], blocks apoptosis by inhibiting mitochondrial p53 accumulation and Bcl-xL activity [29], and inhibits HSP 70 activity [30, 31], but at least in our hands it is an ineffective mitigator (not shown). N-(2-cyclohexyloxy-4-nitrophenyl) methanesulfonamide (NS-398) is an anti-inflammatory COX2 inhibitor with tumor radiosensitizing properties [32–34] that can protect irradiated C3H 10T1/2 fibroblasts from clonogenic cell death [35], but we know of no reports that it can act as a mitigator. In fact, NS-398 diminished stromal cell-mediated mitigation of intestinal radiation damage [36], while COX2 itself has been implicated in TNFR1-dependent LPS-induced radioprotection of intestine [37]. Mitigators active in more than one organ system have been reported previously [38, 39], but our lead compound is exceptional in mitigating not only hARS lethality but also intestinal ARS and pulmonary radiation lethality due to both pneumonitis and fibrosis. Clearly, early intervention can prevent the development of late radiation syndromes.

Radiation tissue damage responses evolve in time and, at least in theory, different targets for intervention may emerge sequentially. Defining how and when different mitigators act is therefore critical for understanding mechanism, rational product development, and combinatorial use. Myeloid cells have long been associated with hARS [19], giving rise to CFU-S on day 8–10 that protect against WBI lethality until such times as the pluripotent hematopoietic stem cell pool recovers [25]. Our observation on the emergence of CD11b^+^Ly6C^+^Ly6G^+^ myeloid cells in bone marrow and peripheral organs soon after WBI is in accord with previous early observations that “neutrophilia” is one of the earliest responses to potentially lethal WBI, which is thought to be related to later (day 4–14) “abortive” rises in cells of this series [16] and CFU-S formation [25]. The drug is known to activate the Wnt pathway in various cells in vitro (Pajonk, in preparation), while in vivo the compounds increase this myeloid response which is essential for hARS mitigation. Further studies are needed to determine if this is a common target for the other tissues that are mitigated, but this seems possible.

Myelopoiesis and myeloid cell mobilization are recognized as features of many pathological situations [40, 41], and play obvious roles in fighting infection, although this cannot be the case in our gnotobiotic mice. The literature on CD11b^+^myeloid cells that co-express both Ly-6G and Ly-6C markers in radiation responses is rather limited. A subset of myeloid-derived suppressor cells (MDSC) has been reported with this phenotype [42], but not all CD11b^+^Ly6G^+^Ly6C^+^ cells have suppressor activity [43]. We suggest that these mitigators not only increase the progenitor pool but polarize and reprogram the developing monocytic and granulocytic lineages [44] after WBI towards an anti-inflammatory phenotype that may make them better at regulating loss and recovery within the stem cell compartment [45]. How these drug-induced early changes in myeloid cell development relate to late pneumonitis and fibrosis is under investigation, but it is of interest that single positive cells with either Ly6G^+^ or Ly6C^+^ derived from the same immature myeloid population have been identified in 2 different models of fibrosis, both with the same protective anti-fibrotic function [43]. This also seems to indicate a common early mechanism that sets the scene for late manifestations of radiation damage.

There is considerable evidence that MDSC emerge after irradiation, and that they can enhance tumor growth, including that of the LLC line used here [46–48]. This makes it even more likely that these drugs are altering the functional profile of the myeloid lineage in addition to expanding the immature subset. The enormous plasticity in the myeloid lineage and their ability to mold their functions in apparently diametrically opposed ways [49], makes tracing the development of the these cells from immediate to late after irradiation of considerable importance.

Currently, there is a dearth of radiation protectors and mitigators for clinical use. The group of compounds in our study may serve as a scaffold for further advancing efficacy in these regards. They had no obvious toxicity even at 40 times the effective dose, and no evidence of being carcinogenic per se or of promoting radiation carcinogenesis, which may not necessarily be the case for all mitigators especially the ones that act through growth promoting pathways. The fact that they can radioprotect as well as mitigate against hARS and have anti- rather than pro-tumor activity, suggests they may be of use in radiation therapy for cancer, which is a promising and tantalizing dualism.

## Conclusions

Members of this group of 4-(Nitrophenylsulfonyl)piperazine molecules are potent mitigators of hARS and probably other ARS and later radiation effects. The broad scope of their action makes them excellent candidates for clinical use as well as in emergency radiation situations.

## Abbreviations

ARS: acute radiation syndrome
CFU-S: splenic colony forming unit
hARS: hematopoietic acute radiation syndrome
HTS: high throughput screen
LLC: Lewis lung carcinoma
LPS: lipopolysaccharide
LTI: local thoracic irradiation
MDSC: myeloid-derived suppressor cells
MST: mean survival time
NPS: 4-nitrophenylsulfonamide
NPSP: 4-(nitrophenylsulfonyl)piperazines
PEC: peritoneal exudate cells
SRM: selected reaction monitoring mass spectrometry
UPLC: ultra-performance liquid chromatography
WBI: whole body irradiation
